# A Second WNT for Old Drugs: Drug Repositioning against WNT-Dependent Cancers

**DOI:** 10.3390/cancers8070066

**Published:** 2016-07-14

**Authors:** Kamal Ahmed, Holly V. Shaw, Alexey Koval, Vladimir L. Katanaev

**Affiliations:** 1 Department of Pharmacology and Toxicology, University of Lausanne, Lausanne 1011, Switzerland; kamal.ahmed@unil.ch (K.A.); holly.shaw@unil.ch (H.V.S.); alexey.koval@unil.ch (A.K.); 2 School of Biomedicine, Far Eastern Federal University, Vladivostok 690922, Russia

**Keywords:** approved drugs, WNT pathway, repositioning

## Abstract

Aberrant WNT signaling underlies cancerous transformation and growth in many tissues, such as the colon, breast, liver, and others. Downregulation of the WNT pathway is a desired mode of development of targeted therapies against these cancers. Despite the urgent need, no WNT signaling-directed drugs currently exist, and only very few candidates have reached early phase clinical trials. Among different strategies to develop WNT-targeting anti-cancer therapies, repositioning of existing drugs previously approved for other diseases is a promising approach. Nonsteroidal anti-inflammatory drugs like aspirin, the anti-leprotic clofazimine, and the anti-trypanosomal suramin are among examples of drugs having recently revealed WNT-targeting activities. In total, 16 human-use drug compounds have been found to be working through the WNT pathway and show promise for their prospective repositioning against various cancers. Advances, hurdles, and prospects of developing these molecules as potential drugs against WNT-dependent cancers, as well as approaches for discovering new ones for repositioning, are the foci of the current review.

## 1. Introduction

WNT signaling is one of the essential pathways involved in animal embryonic development, during which it has numerous roles including the regulation of cell proliferation and differentiation [1]. In the healthy adult tissues however, it is largely inactive, with some exceptions such as the renewal of the gastro-intestinal tract [2], as well as haematopoiesis [3] and regeneration after injury [4]. It is to no surprise then that aberrant activation of this pathway can lead to diseases of neoplastic nature such as cancer [1,5].

**Figure 1:**
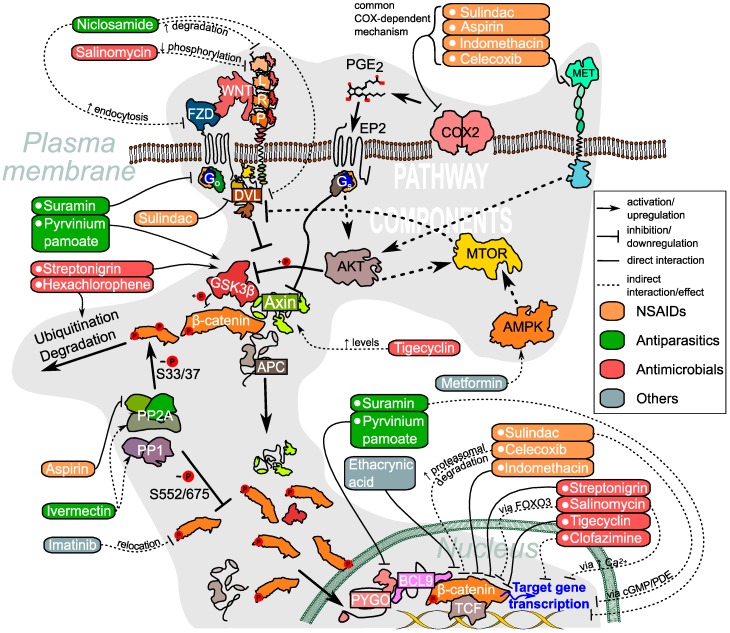
Targets of approved drugs in the context of WNT and related pathways. For detailed descriptions of the activities refer to Table 1 and the corresponding sections in the main text.

The signaling is activated by a family of lipoglycoproteins called WNTs, of which 19 can be found in humans and whose production, secretion and diffusion through tissues is tightly controlled [6]. Upon binding to the FZD family of GPCRs [7,8] (ten homologues in humans), various branches of the WNT pathway can be activated, depending on the ligand-receptor combination and cellular context. They are namely the PCP branch mostly involved in cytoskeleton rearrangement, cell polarity and migration; the WNT/Ca^2+^ branch which is known to promote proliferation and antagonize the canonical pathway; and finally the so-called canonical branch [9]. It is the latter, which is mostly associated with disease and cancer and therefore is the focus of many studies touching upon the WNT pathway [1,10]. Upon WNT binding to the FZD-receptor and one of the two single transmembrane co-receptors LRP5/6, the FZD-coupled G-proteins together with Dishevelled (DVL), a multi-domain scaffolding protein, transduce the signal (Figure 1) [11,12]. As a consequence AXIN, part of the β-catenin destruction complex, is recruited to the membrane [13,14]. The destruction complex is responsible for the phosphorylation of β-catenin and its subsequent degradation. In addition to AXIN, which acts as a scaffold, it also contains adenomatous polyposis coli (APC) and the Ser/Thr kinases casein kinase (CK1) and GSK3β, which in the absence of signaling phosphorylate β-catenin. The phosphorylation of β-catenin results in its ubiquitination and proteosomal degradation [15]. However, upon WNT signaling AXIN is no longer able to form the destruction complex and cytosolic β-catenin accumulates. This leads to its translocation to the nucleus where it exerts its downstream effects by mediating LEF/TCF dependent transcription of WNT-target genes. These include proto-oncogenes such as c-Myc and cyclin D1 [16,17].

To date nearly half of known human tumors show a dysregulation of the WNT signaling pathway [18]. Loss-of-function mutations of APC, which induce adenoma, one of the first steps in the cancerous development, are of the first and probably the best known examples of WNT-dependency in cancer [19]. Since establishing the link between the WNT pathway and tumorigenesis, a broad variety of solid tumors and leukaemias have been shown to either almost entirely or by few subtypes depend on deregulation of the WNT-pathway [18]. Even though the overactivation of the pathway is in some cases due to mutations, in many it is rather the up- or downregulation of pathway components which is the cause. Examples therefor are the upregulation of the WNT receptor FZD_7_ found in certain breast cancers and hepatocellular carcinoma [20,21] or the downregulation of the WNT inhibitory factor 1 (WIF1) found in prostate, lung, breast and bladder cancers [22]. More recently, the WNT-pathway has also been shown to be a player in an emerging field of cancer stem cells (CSC), being involved in their maintenance and survival in certain cancers [23,24], resparking the interest of researchers from various fields in this pathway. In several instances CSCs, thought to be tumor initiating cells, have been demonstrated to be a cause for the occurring drug resistance and metastasis after initial therapy [25]. It is therefore not surprising that in recent years there has been an urgency to discover new drugs targeting this pathway. So far however, no drug targeting the WNT pathway has been approved, and only few have made it into early clinical trials, such as the anti-FZD_7_ antibody vantictumab (NCT01345201) and the PORCN inhibitor LGK974 (NCT01351103) [26].

The traditional drug discovery process has become a costly and time-consuming practice [27,28]. On average, de novo discovery and development of a drug costs about 1.8 billion dollars and it takes around 10–15 years for the drug to reach the market [27]. On estimate, only one in ten drugs entering phase I clinical trials is finally approved by the FDA, and this decreases to one in fifteen for drugs with an oncology indication [28]. Drug repositioning, meaning using known drugs for new purposes, has therefore become an attractive drug development strategy, as it has an attractive risk-versus-reward trade-off compared to other business strategies [29]. Indeed, the advantages of repositioning a drug are multiple: not only has the drug already been used in humans, been tested in various stages of the drug development pipeline and therefore offers knowledge on is safety, pharmacology and toxicology, but also in some cases, later stages of the process such as the manufacturing and formulation can be reused for the new drug product [29]. Especially in oncology, where there is an ever-increasing demand for new therapies, drug repositioning could offer a faster and economically more interesting way of fighting this class of disease [30]. The best-known example of successful drug repositioning against cancer is thalidomide. It was initially used to treat morning sickness but was discontinued after being discovered to cause malformations in new-borns. It was later rediscovered to have anti-cancer properties and is currently FDA-approved for multiple myeloma in combination treatment with dexamethasone [30].

Many WNT-dependent cancers, such as triple-negative breast cancer (TNBC), are unmet medical needs. This makes future drugs against these cancers eligible for receiving the fast track designation granted by the FDA. This designation allows the approval process to be accelerated [31]. We propose that the shortened drug discovery process together with fast track designation makes drug repositioning a promising strategy to win the battle against WNT-dependent cancers, bringing help to patients sooner than later.

This review focuses on approved drugs, which have later been found to modulate the WNT pathway. We highlight their mechanism of action and the range of WNT-dependent cancers these drugs may target in vitro and in vivo. We also correlate these data with the pharmacodynamic and pharmacokinetic parameters established for these drugs, and examine the potential for their repositioning against the WNT-dependent cancers. Finally, we discuss the challenges drug repositioning holds and future possibilities of finding new anti-WNT drugs for cancer treatment.

## 2. Nonsteroidal Anti-Inflammatory Drugs (NSAIDs)

NSAIDs are a class of drugs marketed for their analgesic, anti-inflammatory and anti-pyretic effects. These effects are achieved by the inhibition of the cyclooxygenase (COX) enzymes, COX1 and COX2, involved in the prostaglandin production. They can be classified as non-selective, as is the case for most of the NSAIDs, or selective COX2 inhibitors, as is the case for celecoxib [32].

Apart from their traditional usage, aspirin and other NSAIDs have shown great promise in lowering the incidence of adenomatous polyposis of the colon and reducing the risk of colon cancer. This conclusion is based on several epidemiological studies of the general population and randomized trials [33,34,35,36]. Studied to a lesser extent, the prolonged intake of NSAID has also been linked to a reduction of incidence of various other solid tumors [37,38] such as those of the breast [39,40], lung [41], oesophagus [42], prostate [43], bladder [44], and pancreas [45].

The existence of crosstalk between COX2 and WNT signaling has been demonstrated. Indeed, prostaglandin E2 (PGE_2_) induces β-catenin stabilization, leading to its nuclear translocation, and is able to induce β-catenin/TCF/LEF-reporter activity in colon cancer cells [46,47]. Castellone et al. showed that stimulation of the GPCR EP2 by its ligand PGE_2_ induces activation of Gαs and its association with AXIN, leading to the release of GSK3β from the destruction complex. In parallel, the Gβγ component of the initial heterotrimeric Gs protein activates PI3K/AKT, which in turn inhibits GSK3β by phosphorylation. PGE_2_ therefore has a dual activating effect on the WNT-pathway, and NSAIDs decrease this effect by inhibiting the COX enzymes [46]. The effectiveness of NSAIDs on cancer is in some cases partly due to the COX-inhibitory effect, which leads to lower levels of PGE_2_ production and decreases the β-catenin stabilization [48,49]. In the sections below, we summarize the experimental evidence demonstrating that NSAIDs also target the WNT/β-catenin pathway in COX2-independent manners.

### 2.1. Sulindac

Multiple studies have shown that sulindac is able to increase β-catenin degradation and decrease its nuclear translocation in breast, lung and colon cancer cells in vitro, leading to reduced expression of the β-catenin/TCF target genes [50,51,52]. This was also observed in X-RARalpha-expressing cells, reducing their leukemic phenotype and stemness [53]. A metabolite, sulindac sulfide, has demonstrated WNT/β-catenin signaling blockage and inhibition of proliferation of prostate cancer cells [54].

From a mechanistic point of view, sulindac has been shown to directly affect the WNT-pathway independently of the COX expression. Sulindac is also one of the few WNT-active approved drugs for which the exact molecular targets within the pathway have been described. Sulindac is able to specifically bind to the DVL-PDZ domain, which was proposed to directly inhibit DVL’s interaction with FZDs. Surprisingly, this interaction is characterized by low micromolar K_d_ levels [55], while the IC_50_ of the pathway inhibition by sulindac was found to be almost two orders of magnitude higher [50,52]. This discrepancy might be accounted for by the fact that the most prevalent cell-permeable metabolite of this drug (sulindac sulfide) has a reduced affinity to DVL [55]. Additionally, it has been demonstrated that sulindac is a cyclic guanosine 3′,5′-monophosphate phosphodiesterase (cGMP PDE) inhibitor, which leads to elevated levels of cGMP and activated cGMP-dependent protein kinase (PKG). This in turn results in transcriptional suppression of β-catenin and inhibition of the WNT/β-catenin pathway [56]—potentially more powerfully than the inhibition of DVL-FZD interactions.

Further, sulindac has been shown to effectively reduce tumor growth of colon cancer and intestinal cancer cells in vivo and prevent colon cancer metastasis in mouse models [48,57,58]. The daily doses of sulindac tested in vivo were 20–50 mg/kg, however doses above 20 mg/kg have shown significant toxicity. It should be noted that in order to achieve efficient in vivo inhibition of COX2 by sulindac 10- to 20-fold lower doses are used, indicating a very narrow therapeutic window available to achieve maximal anti-WNT effect. Even at borderline to over toxicity dose of sulindac at 20 mg/kg, plasma levels of the drug were just under 20–40 µM [59], which is somewhat lower than the IC_50_ of COX-independent WNT-inhibition in vitro (50–70 µM) [50,52]. However, this might be compensated by the tissue accumulation of sulindac, exceeding plasma levels by 3–5 fold [59]. Sulindac treatment in mice results in reduced transcript and nuclear β-catenin levels [58,60,61]. In humans, familial adenomatous polyposis (FAP) patients treated with a tolerable dose of 300 mg of sulindac per day for 6 months presented lower adenoma nuclear β-catenin levels than adenomas in non-treated patients [50]. However, at this dose sulindac plasma and tissue levels will not exceed 1–10 µM and therefore WNT-pathway modulation in these patients is likely to be a result of COX2 inhibition.

### 2.2. Aspirin

The chemopreventive effects of aspirin, the only irreversible COX inhibitor, were first reported in a study in 1988 [62] and since, there have been many clinical reports to support such effects [34,36,63]. Diehlmann et al. were the first to demonstrate that aspirin can inhibit β-catenin/TCF transcriptional activity in a luciferase-based reporter assay in colorectal cells lacking COX expression [64]. The drug did not affect the total amount of β-catenin, but the levels of β-catenin phosphorylation (both phospho-S33/S37/T41-catenin and phospho-T41/S45-catenin) were increased, however independently of GSK3β [65]. It was thus hypothesized that aspirin affected β-catenin phosphorylation through inhibition of a phosphatase, which was later discovered to be protein phosphatase A2 (PP2A), being inhibited by aspirin directly [66,67]. Although not yet clearly demonstrated, it is highly likely that aspirin also affects WNT signaling indirectly through other aspirin affected pathways, for example the NF-κB signaling [68]. In vivo models confirm the influence of aspirin on the WNT pathway. In APC^min^ mice, the murine model of FAP, aspirin treatment decreased tumor formation and lowered β-catenin levels [69]. Noteworthily, the amount of data accumulated on aspirin and its effect on various tumors is tremendous and cannot be reviewed here fully. There are multiple studies confirming its effects on WNT signaling and tumor growth in various cancer types both in vivo and in vitro. These findings are excellently reviewed elsewhere [70,71].

In general, lower millimolar levels (~5 mM) of aspirin are needed for the COX-independent in vitro inhibition of the WNT/β-catenin pathway in human cancer cells [64]. To reach similar concentrations in mouse tumor tissue, high doses (ca. 100 mg/kg) of aspirin must be administered [72]. The chemopreventive effect of aspirin in humans, which has already been shown for doses as low as 75 mg/day [73] is most likely due to the COX inhibitory effects. In order to reach the COX-independent WNT inhibitory effects of aspirin, high doses (>10 g/day) need to be administered [74], which however could lead to toxicity and side effects upon treatment, especially in the long-term [75]. Aspirin’s merit as a combination therapy is currently being scrutinized in various trials and retrospective studies, which have already demonstrated its utility in treatment and prevention of notorious WNT-dependent cancers such as breast, colon, prostate and gastric cancers [71]. The first tangible outcome in aspirin repositioning has already been achieved. The US Preventive Services Task Force (USPSTF) recommends a daily low-dose use for individuals with high risk of cardiovascular diseases (CVD) between 50 and 69 years as a mean of both CVD and colorectal cancer chemoprevention.

### 2.3. Indomethacin

Like aspirin, indomethacin has been shown to inhibit proliferation of colorectal cancer cells independently of COX2 expression; however, the mechanisms of action of the two NSAIDS on the WNT pathway were suggested to be different [76]. Indomethacin concentrations of 100–400 µM significantly decrease the TopFlash transcriptional readout in these cells [64], and higher concentrations intensify this inhibition and are accompanied by a decrease in the total β-catenin protein levels [76,77]. The exact reason or details for these effects remain unclear; however, they might partly result from unusual transcriptional regulation of β-catenin, as mRNA levels of β-catenin were significantly lower in the cells treated with indomethacin. The drug has also shown a differential effect on WNT target genes: while cyclin D1 was expectedly downregulated, c-Myc was upregulated. The latter might be the result of a swift onset of apoptosis due to indomethacin treatment [76]. Another input of indomethacin in WNT inhibition is achieved through disruption of the β-catenin-TCF4 complex formation with DNA in colorectal cancer in vitro models [78].

In the rat model of colon cancer, indomethacin (2 mg/kg) was able to reduce tumor formation [79], eliminating nuclear β-catenin staining while leaving cytoplasmic levels unchanged in these tumors [61]. However, it is likely that the anti-WNT effects seen in these studies are mediated through COX inhibition, since these doses result in plasma levels of ca. 10–30 µM of indomethacin [80], far below of what is required for the strong and direct inhibition of the pathway. Applications of higher doses of unmodified indomethacin are unlikely since they are expected to produce acute toxicity (LD_50_ of the drug is around 14 mg/kg for rats).

### 2.4. Celecoxib

The COX2-independent effect of celecoxib was demonstrated by the induction of apoptosis in celecoxib-treated HTC-116 cells, a colorectal cell line lacking the expression of COX2. The effect was proposed to be mediated by inhibition of the WNT pathway, since the drug inhibited the TopFlash reporter and cyclin D1 expression [81]. A second study further showed that in colon cancer cells, celecoxib acted downstream of the β-catenin destruction complex, decreasing TCF1 and TCF4 levels by proteasomal degradation [82]. Complementary to these results, a study demonstrated that in colon cancer cells celecoxib inhibited the c-Met/AKT pathway, resulting in decreased phosphorylation and thus increased activity of GSK3β, leading to an increase in β-catenin phosphorylation [83]. The inhibition of proliferation and the downregulation of the WNT/β-catenin signaling by celecoxib was also demonstrated in glioblastoma and prostate cancer cells [54,84]. In glioblastoma cells, GSK3β phosphorylation was shown to be reduced, leading to β-catenin phosphorylation similarly to the effects observed in the colon cancer cells [84]. Analogous observations were made for hepatoma cells [85] and osteosarcoma cells [86].

Celecoxib also affects CSCs. In colorectal CSCs, celecoxib reduces the chemotherapy-resistant CD133-positive pool, while decreasing WNT activity and expression of stemness markers [87]. In myelogenous leukaemia cells resistant to imatinib, celecoxib sensitized the cells by inhibiting the ABC transporters responsible for drug resistance via WNT and RAS signaling pathways. The study demonstrated downregulation of the WNT activity and pathway components such as GSK3β, β-catenin, LEF1 and TCF4 at protein and mRNA levels [88].

In vivo celecoxib has been shown to prevent the formation of β-catenin accumulated crypts, typical premalignant lesions of colon cancer showing excessive accumulation of β-catenin [89]. Further, celecoxib has been shown to suppress lung cancer cell metastasis in mice through the PGE_2_-GSK3β-β-catenin axis [90]. Like sulindac and indomethacin, celecoxib is able to reduce the amount of β-catenin-positive cells in colon cancer in rats [61]. Finally, celecoxib has been shown to suppress WNT-dependent mammary carcinoma, meningioma and Lewis lung carcinoma in mouse models [91,92]. The doses used in these studies correspond to plasma levels of 3–5 µM, which is somewhat below the average 20 µM required for inhibition of tumor growth. However, the drug can accumulate in tissues to concentrations 2–4 folds higher than in plasma and therefore reach the effective dose [93]. FAP patients treated with celecoxib (400 mg/day) showed a 28% reduction in polyps after 6 months [94], and in 1999 the FDA approved this drug for the indication of FAP but later withdrew the approval due to lack of proof of clinical benefit.

## 3. Antiparasitics

### 3.1. Niclosamide

Niclosamide is an anthelmintic drug approved by the FDA in 1982 for treating intestinal parasite infections, especially cestodes [95]. In addition to its anthelmintic activity, several studies have described anticancer properties of niclosamide. Its anti-proliferative activity has been demonstrated in a wide array of cancer cell lines representative of WNT-dependent cancers: non-small lung carcinoma [96], multiple myeloma [97], hepatoma [98], adrenocortical carcinoma [99], ovarian cancer [100] and glioblastoma [101]. It also suppresses the growth of CD34^+^/CD38^−^ CSCs of acute myeloid leukemia (AML) and CD44^+^/CD24^−^ CSCs of basal-like breast cancer [102,103].

Niclosamide inhibits the canonical WNT pathway with an IC_50_ of 0.2–0.4 µM, similar to that which mediates inhibition of cancer cells growth (0.33–0.75 µM) [104], suggesting that WNT inhibition is involved in niclosamide’s anticancer effects. Several WNT components are involved in the inhibitory action of niclosamide, which vary depending on the cancer subtypes. In osteosarcoma and colorectal cell lines, it inhibits WNT3a-stimulated β-catenin stabilization and LEF/TCF reporter activity through promotion of FZD_1_ endocytosis and downregulation of DVL2 [105,106]. However, niclosamide’s inhibitory effect for breast and prostate cancer cells seems to involve other components of the WNT pathway. Instead of DVL2 downregulation it induces LRP6 degradation associated with inhibition of cell proliferation, invasion and migration of cancer cells [104].

In addition to inhibiting the canonical WNT pathway, niclosamide may mediate its anticancer activities through several other signaling pathways such as NOTCH [107], MTOR [108], NF-κB [97] and STAT3 [96]. This pleiotropy highlights the need of identifying the relevant targets of niclosamide in different tumors.

The anti-cancer effects of niclosamide have also been tested in vivo. When delivered orally at 200 mg/kg, it induces inhibition of tumor growth and impairs metastases formation in colorectal cancer [106]. WNT pathway inhibition by niclosamide in colorectal and basal-like breast cancer models in mice has been demonstrated by immunohistochemical analysis, where lower levels of cytosolic and nuclear β-catenin were observed for the drug-treated mice [103,106]. When delivered directly into the systemic circulation through intra-peritoneal (IP) injection, it resulted in a significant inhibition of breast tumor growth [109], without manifesting any signs of toxicity or mutagenicity [99,101,103]. However, no information is available on its pharmacokinetics after IP or IV injections. In contrast, the poor oral bioavailability of the drug limits its maximal plasma concentrations achievable by that route of administration to 0.1–0.2 µM [106], one order of magnitude below the effective range; other studies have also shown that the plasma concentrations can vary widely due to variable absorption rates by the gastrointestinal tract [110]. These effects limit the anticancer applications of orally delivered niclosamide. Since the safety profile is only available for oral delivery [110], it is only feasible to use it against gastrointestinal tumors so far. One such study has already been launched: the evaluation of efficacy as a treatment of metastatic colorectal cancers patients in a phase 2 clinical trial, using the same approved dose and oral route of administration (NCT02519582) is ongoing.

### 3.2. Suramin

First introduced in 1912, suramin was used for the treatment of African sleeping sickness and river blindness in humans [111]. Despite such a long history and appearance of new agents for the same conditions, suramin is still indispensable in the clinical practice as it remains the only treatment for certain subtypes of the diseases [112].

Suramin has demonstrated a dose-dependent anti-proliferative effect in many human cancer cell lines [113,114,115]. Molecular targets of suramin are numerous. Most relevant for its anticancer effects are the inhibition of binding of many growth factors, e.g., FGF and VEGF, to their cognate receptors [116,117,118], and the folate metabolism [119]. Recently, we have added to this list inhibition by suramin of at least two targets within the WNT pathway, resulting in its complete blockade [120]. While identification of the downstream target is currently ongoing, we investigated the upstream target, since inhibition of the upstream components of the pathway is a promising approach for increasing drug efficiency against WNT-dependent cancers [26,121,122]. We discovered that suramin acted as a competitive inhibitor of GTP uptake by the heterometric G proteins, in turn regulating internalization of the WNT/FZD complexes, which normally serves to amplify the signaling in the WNT pathway [120,123,124]. We have further shown that inhibition of TNBC growth in vitro and in vivo is more efficient at the concentrations ensuring such inhibition (ca. 200 µM) as compared to lower doses.

Since suramin was already proposed as an anti-neoplastic agent in the late-80s, this drug has an extensive record of clinical trials. Although trials with the focus on the WNT pathway targeting are yet to be done, some have already been performed against cancers which strongly rely on the WNT pathway, such as recurrent breast cancer [125,126], metastatic colorectal cancer [127,128], and lung cancer [129]. Surprisingly, in almost all of these trials suramin failed to show any significant improvements, maximally resulting in only moderate positive response reported in two studies [125,129]. In both, suramin was used at small doses (weekly IV perfusions, ca. 100–150 mg per patient) resulting in plasma levels of 30–50 µM; in these cases treatment was not associated with any significant toxicity. In other studies, the doses (weekly IV, 500–700 mg per patient) used produced plasma levels corresponding to that necessary for WNT inhibition (200–250 µM); unfortunately this resulted in significant side effects, primarily of the neurological character with no significant clinical outcomes for tumors [126,127,128]. One of the possible explanations for this might be in the unfavourable pharmacokinetics of suramin. It was found to have poor tissue penetration and retention. Suramin concentrations in most tissues were 2–3 times, and in the tumor (pheochromocytoma in this case) almost six times lower than in plasma [130]. It should be also noted that suramin demonstrated similar negative results against other types of cancers: prostate [131,132,133,134], ovarian [130], urinary bladder cancer [135]. Based on this negative data, the FDA has so far refused approval of suramin for therapeutic applications in oncology [134].

Tackling these limitations of suramin can be achieved by several ways. Since systemic administration of suramin results in multiple toxicities and the gastrointestinal tract has shown poor absorption [111], the repositioning of suramin might be achieved by using new routes of administration to avoid systemic treatment. This has led to a phase 1 clinical trial, testing the efficacy of suramin delivered intravesically for urinary bladder cancer patients [135]. Future directions of suramin applications might be through usage of novel targeted delivery systems to create high local concentrations at the tumor site [136] or synthesis of new structural analogues in order to improve potency and overcome the side effects [137,138,139].

### 3.3. Pyrvinium Pamoate

Pyrvinium pamoate is an anthelminthic drug approved by the FDA [140]. The anticancer activity of pyrivinium is exhibited through inhibition of colon cancer cell motility and proliferation in vitro and suppression of tumor growth in vivo [141].

Inhibition of the WNT pathway by pyrvinium has also been demonstrated in vitro [142,143] and in vivo [141]. Like for most of the repositioned drugs, WNT inhibition by pyrvinium occurs through multiple components of the pathway. Pyrivinium has been demonstrated to act through activation of an isoform of casein kinase 1α (CK1α), part of the WNT pathway destruction complex. In the same work, the authors identified inhibition of pygopus (PYGO), preventing transcriptional activity of β-catenin, as a second impact of pyrvinium on the WNT pathway. These activities are independent of each other and show comparable IC_50_’s [143]. However, another study has failed to recapitulate the effects on CK1α by pyrivinium, and instead suggested that the drug acts through the PI3K/AKT pathway in the manner similar to that described above for celecoxib, decreasing GSK3β phosphorylation at Ser9 and thus enhancing its activity [142]. Several other mechanisms, such as the energy metabolism and STAT3 pathway [144,145], glucose deprivation and hypoxia [146], as well as autophagy [147], have been implicated in the anticancer action of pyrvinium.

In vitro studies have identified pyrvinium to be effective against the WNT pathway and cancer cell proliferation within the high-nanomolar range (50–200 nM). When delivered by its standard oral route, pyrvinium’s bioavailability is virtually zero [148] and therefore cannot be employed for in vivo anticancer studies. Therefore, it was delivered by daily intraperitoneal injections of 1 mg/kg, which were reported to create acceptable peak plasma levels of 150 nM [149]. Using this dose, efficient suppression of the WNT-dependent colon cancer in vivo was achieved [141]. Unfortunately, this dose is borderline with severe toxicity, since any increase resulted in severe toxic effects [149]. Therefore, phase I safety trials should be launched first in order to verify this novel delivery route in patients; no data has been reported so far for any attempts to run such a trial.

### 3.4. Ivermectin

First introduced in 1981 as an anti-parasitic for veterinary applications [150], ivermectin was approved in 1987 for the treatment of onchocerciasis and more recently for lymphatic filariasis in humans [151,152]. It has also been reported to activate chloride channels of nematodes, causing parasite paralysis and death [153].

Ivermectin inhibits proliferation of human colon cancer and lung cancer cells both in vitro and in vivo [154]. The anti-proliferative action, affecting both the bulk tumor cells and CSCs, was linked in this study to inhibition of WNT signaling. The mechanism of this inhibition is rather unusual: ivermectin inhibits C-terminal phosphorylation of β-catenin, overactivating by an unknown mechanism protein phosphatases PP2A and PP1. As a result, the activity of β-catenin as a co-factor in transcription of the WNT target genes is reduced [154].

Ivermectin also has a cytotoxic action due to activation of mammalian chloride channels, similarly to its effects in nematodes [155]. Importantly, the anti-WNT IC_50_ of ivermectin is 5–10 times (~1–2 µM vs. 10 µM) lower than that of its toxic effect against chloride channels. Unfortunately, oral bioavailability of the drug, as for other antiparasitic drugs discussed in this section, is very low. Upon normal oral dosing its plasma levels do not exceed 60 nM. Intraperitoneal delivery at 10 mg/kg in the form of a cyclodextrin conjugate, likely achieving high plasma concentrations, was well tolerated and suppressed growth of colorectal cancer in mouse xenograft studies [154]. Toxicity studies in vivo have also demonstrated a wide therapeutic index for ivermectin [151,156]. The scarcity of data regarding the pharmacokinetics and the safety profile of ivermectin delivered to humans by means other than oral delivery make it compulsory for ivermectin to be tested in safety studies before any further clinical interventions.

## 4. Antimicrobials

### 4.1. Salinomycin

The anticancer properties of salinomycin, an antibiotic potassium ionophore used to treat poultry, were first discovered in a high-throughput screen on breast cancer stem cells [157]. This study demonstrated the ability of salinomycin to reduce the proportion of breast CSCs in vitro and the expression of genes associated with CSC and poor prognosis. Gupta et al. also showed inhibition of mammary tumor growth in mice treated with salinomycin and the promotion of cell differentiation to an epithelial phenotype after treatment [157]. Since then, salinomycin has been shown to inhibit cell growth in the following WNT-dependent cancer cells in vitro: pancreatic [158], endometrial CSCs [159], chronic lymphocytic leukaemia cells [160], breast and prostate cancer cells [161], osteosarcoma CSCs [162], hepatocellular carcinoma cells [163], nasopharyngeal carcinoma cells [164]. It has also showed promising inhibition of growth of gastric tumors, osteosarcoma as well as hepatocellular and nasopharyngeal carcinoma in mice [162,163,164,165].

As the WNT pathway is one of the essential pathways responsible for the survival of CSCs, it has been proposed as one of the targets of salinomycin [160]. Indeed the drug has been shown to downregulate the expression of WNT-target genes such as LEF1, cyclin D1 and fibronectin in vitro [159,160,161] by inhibiting the WNT-induced phosphorylation of the co-receptor LRP6 and inducing its degradation in WNT-overexpressing cells [160,161]. Further it has been shown that salinomycin is able to activate the transcription factor FOXO3, which then disturbs interactions between β-catenin and TCF, inhibiting the transcription of WNT target genes [166]. One additional suggested mechanism of action of salinomycin is the suppression of the canonical WNT-pathway via an increase of intracellular calcium levels, as it has been shown that non-canonical WNT ligands are able to inhibit canonical WNT-signaling by increasing calcium influx [163]. It should be noted that non-WNT related mechanisms of action of salinomycin on cancer cells are multiple, and excellently reviewed elsewhere [167].

The in vitro IC_50_ of salinomycin varies, depending on the source, cell type used and treatment period, between 0.3 and 10 µM. Up-to-date, there is no comprehensive pharmacokinetic study of salinomycin in animals or humans. Similar to the anti-parasitic drugs, salinomycin is normally delivered orally, however this route is unacceptable for anticancer applications due to low bioavailability and therefore resulting low blood and organ levels. It is also shown that after intravenous injection in mice, salinomycin is rapidly metabolized [168] and therefore frequent injections/infusions are likely necessary, though nothing is known regarding the anticancer activities and pharmacokinetics of its metabolites. Prolonged daily injections of 10 mg/kg of salinomycin in mice grafted with nasopharyngeal carcinoma showed no overt toxicity and resulted in a decrease in the tumor burden, and also in reduced levels of LRP6 and β-catenin [164]. Another group has also reported no toxicity and marked tumor reduction concomitant with decreased GSK3β phosphorylation in an osteosarcoma xenograft model in response to 5 mg/kg salinomycin daily [162]. Treatment with 4 mg/kg salinomycin reduced tumor burden in an in vivo model of hepatoma. This also corresponded to a significant shutdown of GSK3β phosphorylation with a concomitant β-catenin decrease [163].

Unfortunately, there is currently little knowledge of the toxicity and pharmacology of salinomycin in humans, as it has never been approved for human use. However, in an uncontrolled clinical pilot study employing salinomycin to treat several patients with various metastatic cancers, metastases regression was observed; in another case of squamous cell carcinoma of the vulva, monotherapy resulted in prolonged progression-free disease. Salinomycin was given at 200–250 µg/kg, which corresponds to the initial concentration in blood plasma of ca. 15–20 µM, agreeing with the mouse dose of 1–2 mg/kg. Acute side effects in all cases were minor and included tachycardia and mild tremors with no observed long-term toxicity [169]. Since then however, there have been no further reports of trials involving salinomycin.

### 4.2. Clofazimine

Our group recently linked the anti-cancer properties of the anti-leprosy drug clofazimine to the inhibition of the WNT pathway. In this study, the library of FDA-approved drugs was screened in silico to identify potential antagonists of the WNT-FZD interaction. Out of the selected higher-scored potential candidates, clofazimine was one of four compounds, which demonstrated significant specific inhibition of the WNT-pathway in vitro when using the TopFlash reporter assay. Despite bioinformatics evidence, the drug was not able to inhibit the WNT-FZD interaction. Instead, it targets the WNT pathway downstream of β-catenin and can inhibit proliferation of TNBC cells [170]. Other potential mechanisms of anticancer effects of clofazimine might be an indirect stimulation of phospholipase A2, resulting in the lysophospholipid-induced apoptotic death [171], or interference of the drug with the respiratory chain [172].

Clofazimine has shown an anti-WNT effect with the IC_50_ in the low-µM range (~3 µM), which is somewhat higher than the usual plasma levels of this drug for anti-leprosy treatment (0.5–1 µM) [173]. However the drug is extremely lipophilic and therefore has a propensity to accumulate in tissues resulting in concentrations of 100–500 µM, which in this case is favourable for the antitumor therapy [170]. While investigation of anti-WNT effects in vivo is now ongoing, these data help to explain previous results of cancer inhibition shown in squamous hepatocellular carcinoma cell cultures [171], in mammary cancer in vivo [174], and in lung cancer in vitro and in vivo [172].

A phase II study has claimed benefits of clofazimine for the indications of unresectable and metastatic hepatocellular carcinoma, where 50% of the patients showed a response or disease stabilization [175]. However, this could not be concluded for the advanced unresectable primary hepatocellular carcinoma, when treated with clofazimine in combination with doxorubicin [176]. Altogether, these studies and the fact that clofazimine is generally considered a well-tolerated and safe drug (its common side-effects include skin discoloration and rashes, palpitations and enterophaties [175,177]) are encouraging for the future repositioning of clofazimine as an anticancer drug directed against highly WNT-dependent tumors such as TNBC. A future challenge will be managing and discovering the effect of clofazimine, when used at high doses for long-term oncology therapy.

### 4.3. Other Antimicrobials

Salinomycin and clofazimine are not the only antimicrobial drugs in the spotlight for repositioning against WNT-dependent cancers. In this section we review three other compounds approved for human use, which do not benefit from extensive records in scientific literature but have shown promise for targeting the WNT pathway.

Tigecyclin, a tetracycline derivative, inhibits human cervical cancer cell growth in vitro and in vitro, especially when combined with the well-known chemotherapeutic paclitaxel. It decreases both cytoplasmic and nuclear levels of β-catenin and decreases transcription of the WNT-target genes, while increasing the levels of AXIN1 [178].

The antitumor antibiotic streptonigrin was in anticancer trials until 1977 but was discontinued as the toxic effects outweighed therapeutic benefits. The drug’s original mechanism of action was mostly due to the induction of DNA damage [179,180]. It has recently been demonstrated that the anti-neoplastic effect might also be achieved through the inhibitory effects of streptonigrin on the β-catenin/TCF complex formation with DNA. However, it seems that this drug has additional targets since suppression of GSK3β phosphorylation and decrease in β-catenin were also observed [181].

Hexachlorophene, a disinfectant previously used as a bacteriostatic skin cleanser, has demonstrated WNT/β-catenin pathway inhibition by promoting degradation of β-catenin through the ubiquitin ligase SIAH1 in colon cancer cells and EBV-infected B-lymphoma, as well as inhibition of cell proliferation in colon cancer cells [182,183].

## 5. Additional Selected Compounds

### 5.1. Metformin

Metformin was originally developed as an antidiabetic drug, stimulating the adenosine monophosphate activated protein kinase (AMPK). It was approved by the FDA in 1995. The anticancer effects of metformin have been demonstrated by population-based retrospective studies that reported a decrease in the cancer incidence and a better cancer prognostic outcome in diabetic patients diagnosed with cancer treated with metformin, in comparison to diabetics diagnosed with cancer while not treated with metformin [184,185].

A recent study revealed that anti-proliferative actions of metformin are also associated with the indirect inhibition of the WNT pathway. Surprisingly, its effects are mediated through its original target—AMPK, which then employs the MTOR signaling pathway to promote the ubiquitination and proteasomal degradation of DVL3, one of the principal WNT transducers [186]. This is very encouraging as it means that the drug can be used at its normal dose to exert its anti-WNT effects, and indeed the doses of metformin reported in the study corresponded to those found for AMPK activation in human tissues [187]. However, AMPK is a multi-faceted target, acting not only through the MTOR pathway, but also involved in regulation of the mRNA translation machinery [188]. In addition, metformin’s activities may involve perturbations of tumor metabolism and may be mediated by immunomodulatory mechanisms, sustaining the anticancer immune response [189]. Overall, the anti-proliferative action of metformin in cancer cells has been shown in vitro against lung, pancreatic and gastric cancers [190,191,192] and both in cell lines in and in preclinical models of hepatocellular carcinoma and in ovarian CSCs [193,194].

As the discovery of the anticancer effect of metformin in 2013 was based on clinical data from more than 5000 breast cancer patients (1013 out of them were taking metformin), the results are essentially equivalent to those of a large-scale Phase III clinical trial. This, in combination with no need to significantly escalate the dose or change the delivery route of the drug, expectedly sparked immediate attention of clinicians to metformin. There are 55 clinical trials that have been launched since then, testing the anticancer activity of metformin against a large diversity of cancers in various phases with different endpoints. Any definitive results from these trials should be expected in a few years from now and for details one may consider this excellent review [188].

### 5.2. Imatinib

Imatinib, known under the trade names of Gleevec/Glivec, is a tyrosine kinase inhibitor targeting BCR/ABL, which is the primary target in chronic myeloid leukemia, and some receptor tyrosine kinases (PDGFR, c-KIT) important in gastrointestinal stromal tumors [195]. Its tyrosine inhibitor function has shown to also affect the WNT/β-catenin signaling in anaplastic thyroid carcinoma cells in a c-ABL dependent manner. Imatinib-treated cells have reduced transcription of the WNT target genes such as cyclin D1. Imatinib also reduced β-catenin levels, inducing its relocation from nucleus to the plasma membrane, decreasing cell invasiveness [196]. In colon cancer cells, imatinib has shown similar effects indicating that it can be efficient against different WNT-dependent cancers [197].

### 5.3. Ethacrynic Acid

The WNT-inhibitory effects of the loop diuretic ethacrynic acid (EA) were first discovered in a library screen containing 960 FDA approved drugs. EA inhibited the TopFlash reporter in a dose-dependent manner and was further demonstrated by co-immunoprecipitation studies to target LEF1 and destabilize formation of its complex with β-catenin [198]. In patient-derived chronic lymphocytic leukaemia cells, EA reduced expression of the WNT-target genes such as fibronectin, cyclin D1 and LEF1 [198]. Another study has additionally shown that treatment of myeloma cells with EA results in decreased levels of β-catenin, which points toward existence of several inputs of this drug in WNT signaling inhibition. In vivo EA alone has shown excellent promise and was able to inhibit myeloma growth and prolong survival in mice more efficiently than lenalidomide, the drug currently used in patients with multiple myeloma [199,200].

In humans the maximum dose of EA when administered by intravenous injection is 100 mg/day, which results in plasma levels of around 30 µM [201]. This corresponds to the WNT inhibitory doses used in the in vitro studies [198,199]. In mice, the oral dose of 450 µg/day should result in plasma levels close to those in humans mentioned above, meaning that inhibition of the tumor growth may also be feasible in humans, however no reports of such a study currently exist.

### 5.4. Riluzole

Several studies reported that in a significant number of melanoma cases the WNT ligand responsible for the invasiveness and metastasis is non-canonical WNT5A which is known to suppress canonical signaling and function through other branches of the WNT signaling [202]. This makes these subtypes of melanoma the only known case of cancer which does not benefit from the elevated levels of canonical WNT signaling. On the contrary, elevated β-catenin levels in corresponding models of the disease have been associated with reduced cell proliferation and improved patient survival, which are the result of induction of cell differentiation [203]. This prompted the screening aimed at finding WNT pathway enhancers, in which the FDA-approved riluzole, a therapeutic for amyotrophic lateral sclerosis, was identified. Further testing of riluzole on melanoma cells in vitro showed that it is indeed able to enhance the ability of WNT3a to inhibit cell proliferation and promote pigmentation. In vivo riluzole was able to decrease metastases formation in mouse models. The authors further identified the glutamate receptor GRM1, a known indirect target of riluzole, to be a regulator of WNT/β-catenin signaling, linking inhibition of GRM1 by the drug to enhancement of the WNT pathway [204]. Patients with GRM1-positive metastatic melanoma were enrolled in a “Phase 0” clinical trial, preliminarily assessing the effects of treatment with riluzole. The study has shown positive dynamics both in regard of pathological responses and biomarkers (pERK and pAKT), favouring further studies in this direction [205]. It should be noted that repositioning of positive WNT modulators is not only attractive against melanoma, but can be extended into the fields of regenerative and anti-ageing medicine where the WNT pathway is in charge of tissue renewal and may be employed to achieve better outcomes [26,206].

## 6. Challenges and Future Directions for Repositioning WNT Inhibitors in Cancers

As we may conclude, search of the WNT pathway inhibitors among the existing drugs is an idea which excites many minds in the broadly defined field of translational research. Many of them are attracted by the fast-tracking of the results into the clinic, as well as by the usual sheer availability of the mass-produced drug compounds and information on their various aspects such as solubility, metabolic stability and toxicity. This work has already resulted in a considerable amount of promising results reviewed here (Table 1 and Figure 1). However, use of the approved drugs is a double-edged sword, and here we would like to discuss some of the emerging challenges and problems of this approach.

Challenges of the first type are not unique for the WNT signaling but instead concern any attempts to reposition an existing drug for a new purpose. These obstacles of general nature are as follows (also see reviews [29,207]):

- Frequently, for a novel application the drug is required at a higher dose, for an extended treatment period or with a different formulation as compared to the conventional indication in order to demonstrate a significant effect. This may result in unexpected side effects, jeopardizing the idea of the “fast tracking” of the compound due to necessity of a full-scale preclinical and clinical investigation.
- Intellectual property difficulties due to multitude of patents.
- Drug-drug incompatibility: acceptable levels of adverse effects for one application might make the compound useless or uncompetitive for another purpose, as well as incompatible with other treatments for the purpose.
- Different legal statuses of the drug in various countries, e.g., dependence on the region where the disease is widespread or on the socioeconomical status of the population.
- Multiple and controversial mechanisms of novel action, resulting from superposition of the original drug mechanism with the novel one(s).

In addition to that, there are certain challenges, which are specific for drug repositioning for targeting the WNT pathway in cancer:

- The WNT pathway is complex. Many components of the signaling are shared with other pathways, generating cross-talks of varying intensities. Therefore, it is sometimes difficult to clearly distinguish direct influence of the drug on the WNT pathway from its effects on the intersecting pathways.
- Identification of the molecular target is a complicated process, and it is frequently omitted by researchers. Out of the 16 drugs we reviewed here, only EA, suramin, sulindac, pyrvinium pamoate and indomethacin were shown to directly affect identified components of the WNT pathway. Additionally, metformin is known to affect WNT signaling via a cross-talk from its original target AMPK. Delaying the unequivocal identification of the novel molecular target makes it problematic to optimize the drug and evaluate of the scope of its anticancer applications.

It should be also noted that the WNT pathway is not exclusively employed during development or overactivated in cancer. In adults many healthy tissues rely on it for renewal and homeostasis maintenance, most notably the intestine, haematopoietic system, hair, bones and skin. Therefore one might expect adverse reactions in all these organ systems, which has indeed been observed for many WNT-targeting compounds upon attempts to push them into the clinics. The intestine seems to be the most vulnerable in this regard, causing the failure of many anti-WNT agents. As examples, XAV939 and LGK974 result in severe intestinal toxicity in mice, while OMP18RP induces abdominal pain, constipation and diarrhea in patients [208,209].

An interesting and promising direction is the modification of approved drugs for novel diseases. In general, this approach dictates the necessity of full-scale de novo trials, however it still might be considered a future path in drug development. Although the data accumulated for the parent drug cannot be used directly, they will still serve as a strong guide and facilitator in the drug development process. Moreover, frequently there are libraries of the drug derivatives used during the development of the original compound, already available for testing. Efficacy of novel derivatives may allow to overcome many problems we described above for parent molecules, such as multiplicity of mechanisms, dose elevations, and not to forget the hurdles involving the intellectual property.

Of the drugs reviewed here, only some were subjected to medicinal chemistry optimization. Derivatives of niclosamide were synthesized with better metabolic stability without compromising WNT inhibition [210]. In another study, >40 ethacrynic acid derivatives were reported, the best ones with enhanced WNT inhibitory action were further found to inhibit growth of chronic lymphocytic leukaemia cells [211]. A salinomycin-based drug VS-507 is part of the research portfolio of Verastat, a company whose main focus is the development of anti-CSC therapies [169]. Additional noteworthy attempts to improve salinomycin aimed at reducing its toxicity [212] and improving its potency [213]. There are also reports on derivatives of NSAIDs lacking COX inhibition and showing inhibitory effects on cancer cell lines and tumors in rodent models [57,214,215]. Other derivatization attempts were aimed at overcoming the side effects of NSAIDs, producing nitric oxide releasing aspirin (NO-ASA) and phospho-sulindac, with improved potency and lower gastro-intestinal adverse reactions in mice [216,217].

## 7. Concluding Remarks

WNT signaling is one of the developmental pathways [216], whose reactivation in many adult tissues underlies oncogenic transformation. Although no drugs against this pathway are yet on the market nor even in advanced clinical trials, the demand for such drugs is urgent. First medications targeting the hedgehog signaling pathway—another embryogenic pathway responsible for various types of adult cancers, previously also evading drug discovery efforts, have recently been approved [26,217]. This success should inspire researchers developing the anti-WNT agents to continue their quest. Clearly, all possible drug discovery approaches (antibodies, de novo screening of synthetic small molecules, rational drug design and in silico screening, natural products, etc.) are welcome in this task [218]. Repositioning of existing drugs for the new indication of treating WNT-dependent cancers is one of such avenues. Examples discussed in this review illustrate the achievements and remaining hurdles on this path, and reflect our cautious optimism that continuation of it may eventually ensure appearance of first-in-class medicines to treat devastating diseases hijacking the WNT pathway for their progression.

## Figures and Tables

**Table 1:** List of drugs known to modulate the WNT pathway in cancer.

Drug Category	Drug Name	Mode(-s) of Action of WNT Inhibition	Outcome in Vitro	Outcome in Animal Models	Clinical Results
**NSAID**	Sulindac	PGE_2_/COX-dependent	Inhibition of proliferation in breast, lung and colon cancer cell lines	Reduction in tumor growth and metastasis in colon (xenograft and chemically-induced and intestine) while decreasing β-catenin levels	Reduction in β-catenin nuclear staining of adenomas in familial adenomatous polyposis (FAP) patients treated for 6 months
		Direct binding to DVL3 and likely inhibition of interaction with FZDs.			
		Transcriptional suppression as a consequence of direct cGMP PDE inhibition			
	Aspirin	PGE_2_/COX-dependent	Proliferation inhibition in virtually any WNT-dependent cancer	Decreased tumor formation in FAP murine model with concomitant decrease in tumor β-catenin levels	Retrospective studies, especially for colon cancer prevention
		Inactivation of PP2A and phosphorylation of β-catenin			Multiple trials for combination therapy and chemoprevention
		Cross-talk with other aspirin-affected pathways (e.g., NF-κB)			Recommended for CRC prevention in people between 50–69 years old
	Indomethacin	PGE_2_/COX-dependent	Inhibition of growth in colorectal cancer cell lines	Reduced tumor burden in chemically induced colon cancer; reduced β-catenin nuclear staining	No data available yet
		β-catenin degradation through transcription inhibition			
		Disruption of β-catenin/TCF4 complex			
	Celecoxib	PGE_2_/COX-dependent	Impaired proliferation in colorectal cancer, hepatoma, osteosarcoma, glioblastoma and prostate cells lines; Reduction of CD133^+^ colon cancer stem cells; sensitization of imatinib-resistant leukaemia cells	Inhibition of β-catenin-positive premalignant lesions in the mice colon and in rat colon cancer model	Reduction of polyps in FAP patients after 6 months of treatment
		Promotion of TCF1 and TCF4 proteasomal degradation		Prevention of lung cancer metastasis in mice	FDA approval for the prevention of cancer in FAP patients retracted due to lacking proof of clinical benefit
		c-Met/AKT pathway cross-talk promoting GSK3β phosphorylation		Suppression of mammary carcinoma and Lewis lung tumor	
**Antiparasitics**	Niclosamide	Promotion of FZD_1_ endocytosis	WNT pathway inhibition is associated with reduction of cell numbers in osteosarcoma, colorectal, breast and lung cancer cell lines; also effective against hepatoma, glioblastoma, andrenocortical and ovarian cancers	Lowers β-catenin levels in mice models of colorectal and basal-like breast cancers	No data available yet
		DVL2 downregulation			
		LRP6 degradation			
	Suramin	Inhibition of target gene expression via unidentified downstream target	Tested and found effective against virtually all WNT-dependent in vitro cancer models	Extensive record of in vivo studies involving WNT-dependent cancers	Enrolled in multiple trials; mildly effective or ineffective in a combination therapy against breast cancer; reported multiple toxicities when used in doses comparable to WNT-inhibitory ones
		Inhibition of WNT endocytosis through direct inhibition of heterotrimeric G proteins			
	Pyrvinium pamoate	Direct CK1α activation	Efficient against colon cancer	Inhibits tumor growth in colon cancer model	No data available yet
		Pygopus inhibition			
		Direct activation of GSK3β			
	Ivermectin	Deactivation of β-catenin by reduced C-terminal phosphorylation through overactivation of PP2A and PP1 phosphatases	Anti-proliferative for colon (including stem cells) and lung cancers	Reduction of tumor growth in the xenograft models of the colon cancer with reduced WNT markers levels in the tumors	No data available yet
**Antimicrobials**	Salinomycin	Inhibits LRP6 phosphorylation and induces its degradation	Reduction of cancer stem cells in osteosarcoma and breast and endometrial cancers. Anti-proliferative for many WNT-dependent cancer cell lines, e.g., hepatocellular carcinoma, CLL, pancreatic, nasopharyngeal, breast and prostate cancers.	Inhibition of growth of gastric tumors, osteosarcoma, hepatocellular carcinoma and nasopharyngeal carcinoma with signatures of WNT signaling deficiency (reduction of LRP-6 and β-catenin; decreased GSK3β phosphorylation)	Clinical uncontrolled pilot study on several cases with metastatic cancers with positive dynamics such as metastasis regression observed. Minor acute toxicity reported (tachycardia and mild tremors)
		Activation of FOXO3, leading to interrupted β-catenin/TCF interactions			
		Likely inactivation of canonical WNT pathway by increasing Ca^2+^ levels			
	Clofazimine	Exact mechanism is unknown; is likely involved in inhibition of transcription complex	Growth inhibition of squamous hepatocellular carcinoma and lung cancer	Growth inhibition of lung and mammary cancer growth	Several combination and monotherapy studies on hepatocellular carcinoma with mild positive results.
	Tigecyclin	Decrease in β-catenin protein	Cervical cancer cell growth inhibition	Cervical cancer xenografts growth inhibition	No data available yet
		Increase in AXIN1			
	Streptonigrin	Direct inhibition of β-catenin/TCF binding to DNA	Growth inhibition of β-catenin-dependent colorectal and gastric cancer cell lines	No data available yet	No data available yet
		Suppression of GSK3β phosphorylation			
	Hexachlorophene	SIAH1 mediated degradation of β-catenin	Inhibition of colon cancer and B lymphoma cells growth	No data available yet	No data available yet
**Others**	Metformin	AMPK-induced proteasomal degradation of DVL3 through MTOR crosstalk	Anti-proliferative in lung, pancreatic, gastric cancer, hepatoma and ovarian cancers	Inhibit tumor growth in hepatocellular carcinoma and ovarian xenografts	Retrospective study of more than 5000 breast cancer patients showing clear survival benefits
					55 trial launched, no conclusive data yet
	Imatinib	Reduction of β-catenin and WNT-pathway target genes	Anti-proliferative in thyroid carcinoma cells and colon cancer	No in vivo WNT effects were reported yet	Approved for use for multiple cancers
		Relocation of β-catenin to plasma membrane			
	Ethacrynic acid	Inhibition of LEF1/β-catenin complex formation	Anti-proliferative in CLL and myeloma cells	Reduced tumor growth for myeloma in mice	No data available yet
		β-catenin reduction			
	Riluzole	Inhibition of the pathway through target receptor GRM1	Induces melanoma cells differentiation and reduces proliferation	Inhibits metastases	A pilot study assessed safety and efficacy of the compound through biomarkers (pERK and pAKT).
